# The importance of early detection of calcifications associated with breast cancer in screening

**DOI:** 10.1007/s10549-017-4527-7

**Published:** 2017-10-17

**Authors:** J. J. Mordang, A. Gubern-Mérida, A. Bria, F. Tortorella, R. M. Mann, M. J. M. Broeders, G. J. den Heeten, N. Karssemeijer

**Affiliations:** 1 Department of Radiology and Nuclear Medicine, Radboud university medical center, Nijmegen, The Netherlands; 2 Department of Electrical and Information Engineering, University of Cassino and Southern Lazio, Cassino, Italy; 3 Department for Health Evidence, Radboud university medical center, Nijmegen, The Netherlands; 4 Dutch Reference Centre for Screening, Nijmegen, The Netherlands; 5 Department of Radiology, Amsterdam Medical Center, Amsterdam, The Netherlands

**Keywords:** Mammography, Breast cancer, Screening, Invasive ductal carcinoma, Ductal carcinoma In Situ, Calcifications

## Abstract

**Purpose:**

The aim of this study was to assess how often women with undetected calcifications in prior screening mammograms are subsequently diagnosed with invasive cancer.

**Methods:**

From a screening cohort of 63,895 women, exams were collected from 59,690 women without any abnormalities, 744 women with a screen-detected cancer and a prior negative exam, 781 women with a false positive exam based on calcifications, and 413 women with an interval cancer. A radiologist identified cancer-related calcifications, selected by a computer-aided detection system, on mammograms taken prior to screen-detected or interval cancer diagnoses. Using this ground truth and the pathology reports, the sensitivity for calcification detection and the proportion of lesions with visible calcifications that developed into invasive cancer were determined.

**Results:**

The screening sensitivity for calcifications was 45.5%, at a specificity of 99.5%. A total of 68.4% (*n* = 177) of cancer-related calcifications that could have been detected earlier were associated with invasive cancer when diagnosed.

**Conclusions:**

Screening sensitivity for detection of malignant calcifications is low. Improving the detection of these early signs of cancer is important, because the majority of lesions with detectable calcifications that are not recalled immediately but detected as interval cancer or in the next screening round are invasive at the time of diagnosis.

## Introduction

The purpose of breast cancer screening is to detect cancer as early as possible [1, 2]. The earliest signs of non-palpable breast cancer are calcifications, which are usually associated with ductal carcinoma in situ (DCIS) but can also be present in invasive cancers [3]. In screening programs, between 12.7 and 41.2% of women are recalled with calcifications as the only sign of cancer [4–7].

The Breast imaging reporting and data system (BI-RADS) [8] was designed by the American College of Radiology to standardize breast imaging reporting and to provide clarity on the interpretation of breast imaging studies. A set of guidelines is supplied in the BI-RADS atlas for the interpretation of calcifications, aiding the radiologist in distinguishing suspicious calcifications from typically benign changes, such as vascular and skin calcifications. It is recommended to recall patients with suspicious calcifications for further clinical assessment, such as a biopsy [8, 9]. This can inadvertently lead to false positive outcomes, since calcifications associated with benign disease often look suspicious.

The vast majority of cancers detected by calcifications are DCIS, of which < 20% are low grade [10, 11]. In the discussion about the pros and cons of breast cancer screening, detection of low-grade cancers is generally regarded as overdiagnosis [11, 12], since the detection of these cancers does not impact mortality reduction [13]. However, it is not possible to radiologically distinguish calcifications associated with low-grade DCIS from more aggressive forms (grade II and III) in mammography, while these forms should be detected as early as possible [13–15]. Therefore, radiologists in breast cancer screening are instructed to recall all suspicious calcifications. However, in practice, especially in countries where screening programs pursue very low recall rate (i.e., the percentage of screening exams that are recalled in screening) [16], radiologists do not recall patients with calcifications without the reasonable likelihood that they represent DCIS. In such scenario, interpretation of calcifications depends more on the training, experience, and skill of the screening radiologists in a dual reading setting.

There are many studies in which screening mammograms have been retrospectively evaluated to determine the sensitivity of the screening in detecting breast cancer [17–28]. For instance, Vitak [19] re-examined screening exams performed prior to the diagnosis of 544 interval cancers, i.e., cancers diagnosed between screening exams usually due to symptoms, reporting that 25% of these patients could have been recalled based on the screening mammogram. Destounis et al. [25] have found that cancer was visible in 31% of 318 exams prior to a later screen detection, while Burhenne et al. [28] have shown that cancer was visible in 67% of 427 such cases. Broeders et al. [24] have shown that half of 234 screen-detected and interval cancers were already visible on a prior exam. Other studies have reported that around 40% of screen-detected cancers could be detected on a previous exam [18, 23, 29]; however, note that all the aforementioned studies have been performed on screen-film mammography results and cannot be directly compared to digital mammography, the current acquisition standard in breast cancer screening. Several studies have shown that recall rates and cancer detection rates resulting from suspicious calcifications differ significantly between screen-film or digital mammography [6, 30]. Studies by Knox et al. [26] and Weber et al. [27], in which digital mammography screening performance was assessed, have reported that between 10.5 and 31% of interval cancers were missed in screening.

In most of these studies, no distinction was made between soft tissue lesions and calcifications, and generally only interval cancers were evaluated to determine false negatives. However, cancers that were detectable but missed in a prior screening can also be considered as false negatives. In this study, we include false negatives on prior mammograms of both screen-detected and interval cancers. We focus on earlier detection of calcifications, which can prevent the development of invasive disease. A better understanding of this phenomenon is not only relevant in relation to interval cancers, but also to screen-detected cancers, independent of the threshold used for recall.

The purpose of this study is to estimate how often malignant calcifications are not detected in a population-based screening program with double reading, and to determine the proportion of invasive cancers detected by the presence of calcifications that were not recalled in the previous screening round. For this purpose, an accurate assessment of the presence of calcifications in mammograms was performed in a large screening cohort using a computer-aided detection (CAD) system, in combination with visual inspection by an experienced radiologist. This provided a solid ground truth for the analysis. This also allowed us to accurately assess the sensitivity of screening for calcifications associated with breast cancer, in programs equipped with modern digital mammography systems.

## Materials and methods

### Materials

All data used in this study were collected from a single region of the Dutch Breast Cancer Screening Program (Bevolkings Onderzoek Midden-West, The Netherlands). In the Dutch Breast Cancer Screening Program, women between the age of 50 and 74 are biennially invited for a screening exam. This database contained all of the available screening exams, consisting of medio-lateral oblique and cranial–caudal views of the left and right breasts, from all screened women between 2003 and 2014. All images were acquired with full-field digital mammography systems (Hologic, Bedford, Massachusetts, United States). After acquisition, two radiologists independently assessed the mammogram and scored both breasts according to the BI-RADS. When there is a discrepancy between scores, a consensus meeting is held and when no consensus is reached, a third radiologist breaks the tie [31]. Women with BI-RADS 4 or 5 are recalled for further investigation.

**Fig. 1:**
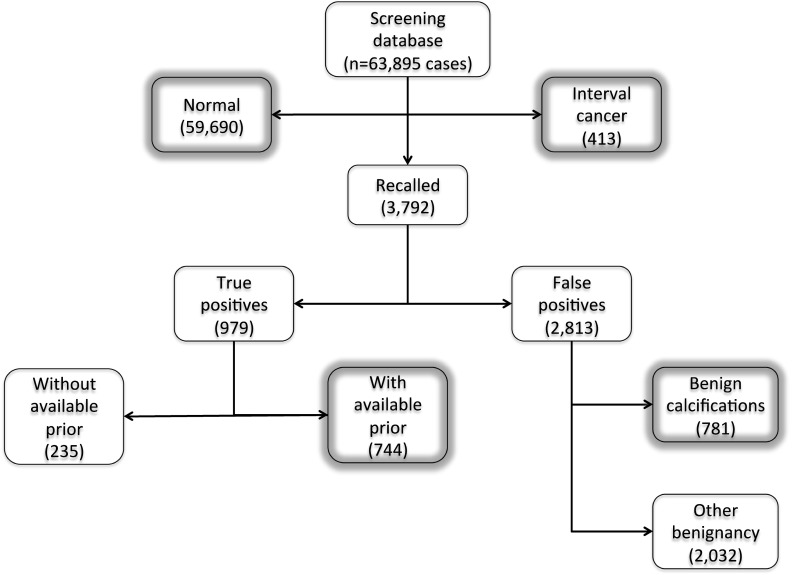
Overview of the breast cancer screening database used in this study, with data from 2003 to 2014. In this study, we include 170,878 screening exams from 63,895 women. Boxes with a gray glow were included in the ground truth for the evaluation

An overview of the screening database is shown in Fig. 1. During the study period, 63,895 women (age 59 ± 7) participated in the screening program (with a total of 170,878 screening exams). In 59,690 women, no abnormalities were found in any of their screening exams. A total of 3792 women were recalled for diagnostic follow-up, of whom 979 had breast cancer. The remaining 2813 recalled women were false positives, of whom 781 were recalled based on calcifications only. In 413 women, an interval cancer was found between screening exams.

### Methods

To construct the ground truth, we identified all women in our database with a pathologically proven invasive and non-invasive breast cancer for whom a negative prior screening exam was available in addition to the screening mammogram that led to the detection of the cancer. Furthermore, we identified all negative screening exams prior to interval cancers, and all exams that were recalled solely based on calcifications but were pathologically proven to be benign. Exams of women without any abnormalities were included as well. We excluded women who were recalled multiple times (*n* = 49) or those who were recalled and later diagnosed with an interval cancer (*n* = 11), to avoid complications in the analysis. The data included in this study are highlighted in Fig. 1.

To determine the ground truth regarding the presence of mammographically visible calcifications associated with cancer, we used radiology and pathology reports and a retrospective review of negative prior exams by a radiologist with more than 25 years of experience in reading mammograms and more than 15 years certified as a screening radiologist. To reduce the subjectivity and workload of the radiologist, a state-of-the-art CAD system [32, 33], operating at its highest sensitivity, was first applied to all mammograms of women with a screen-detected or an interval cancer. The CAD system was developed in house, but we took care that mammograms used to train the system were not included in the study dataset to avoid bias. This training set comprised less than 1% of the total number of normal exams in the screening database and less than 3% of the screen-detected cancers with a prior exam. Before the radiologist inspected the cases, an initial visual inspection by a researcher with experience in reading mammograms was carried out to exclude false positive CAD findings that were obviously not related to the recalled malignancies such as detected noise or vascular calcifications. The radiologist visually inspected the remaining exams to determine whether they were related to the later diagnosis of screen-detected or interval cancer. Prior exams of screen-detected cancers were visually inspected together with the subsequent screening mammogram and radiology reports in which the cancer was detected. For the interval cancers, diagnostic mammograms and radiology reports were not available because the anonymized data in the database could not be linked to the hospitals where the assessment took place. Only the laterality of the interval cancer was known. The visual assessment was performed on a 12MP Coronis Uniti mammography monitor (Barco N.V., Kortrijk, Belgium).

In the constructed database, the number of exams with detectable calcifications was determined for the false negative exams, which contained visible calcifications related to the cancer prior to the diagnosis of a screen-detected cancer (*n*_prior_) or an interval cancer (*n*_interval_). The number of exams with detectable calcifications was also determined for the true positive screening exams, which did not have visible calcifications in the prior exam (*n*_SD_). In this way, each woman with malignant calcifications was represented only once in the series. The screening sensitivity for detecting calcifications associated with cancer was calculated as follows:

$${\text{Sensitivity}} = \frac{{n_{SD} }}{{n_{\text{SD}} + n_{\text{prior}} + n_{\text{interval}} }}100\% .$$

The proportion of invasive and non-invasive cancers at the time of detection was calculated to assess how often women with calcifications at the site of the detected cancer were diagnosed with invasive cancers. The invasive status of each cancer was obtained from the pathology reports. Finally, to analyze tumor size at the time of detection, the tumor stage (as T1, T2, or T3 [34]) was also collected for all invasive cancers.

## Results

**Fig. 2:**
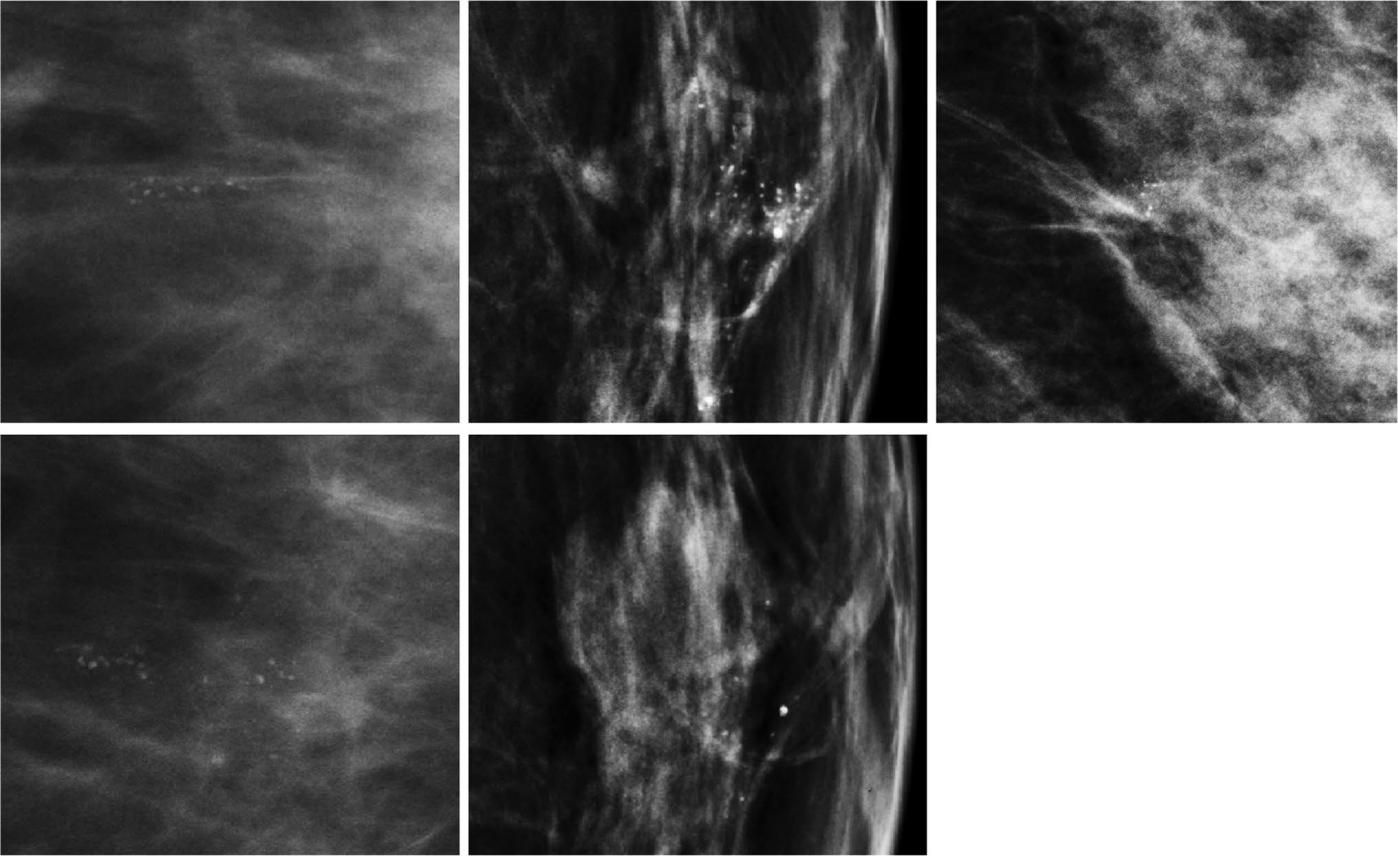
Examples of calcifications detectable on prior mammograms. The top row contains examples of exams prior to screen-detected cancer with calcifications (first column), prior to a soft tissue lesion (second column), and prior to an interval cancer (third column). In each exam, a radiologist identified detectable calcifications related to the cancer. In the bottom row, the same locations are shown as above, focusing on where the cancer and soft tissue lesions were detected (first and second column, respectively)

Exams of all 744 screen-detected cancers and 1157 exams obtained prior to a screen-detected or interval cancer were processed with the CAD system. In 536 of the 1157 prior exams, the CAD system detected at least one instance of calcifications. Of these, the researcher classified 112 as obvious false positives that were not related to the cancer. CAD findings in the remaining 434 exams were visually inspected by the radiologist, who determined that 177 exams contained calcifications related to cancer. Figure 2 shows three examples of non-recalled screening exams with calcifications: (1) prior to a screen-detected cancer with calcifications, (2) prior to a soft tissue lesion, and (3) prior to an interval cancer.

By including the calcifications detectable in prior exams, we identified 325 exams with calcifications associated with malignancy in our dataset. Of these exams, 45.5% (*n*_SD_ = 148) had calcifications that were only detectable at the time of the recall. The remaining 54.5% (*n*_prior_ + *n*_interval_ = 177) were detectable in the previous negative mammograms: 36.3% (*n*_prior_ = 118) on exams prior to a positive screening exam and 18.2% (*n*_interval_ = 59) on exams prior to an interval cancer. An overview of the distribution of these prior exams is shown in Table 1. These numbers were used to compute the sensitivity for the detection of calcifications associated with breast cancer in digital mammogram screening; the screening sensitivity for malignant calcifications was calculated to be 45.5%. The specificity of malignant calcification detection, which was calculated from 166,673 exams without abnormalities and 781 false positive exams with calcifications, was 99.5%. This means that only 0.5% of the exams were falsely recalled in screening based on calcifications alone.

**Table 1:** Overview of the prior exams included in the ground truth. A CAD system was applied to each prior exam. Only the exams with calcifications found in the same region as the cancer were visually assessed by a radiologist, who identified calcifications related to cancer

Assessment of calcifications related to cancer in prior exams	Prior exams available	Prior exams assessed by radiologist	Prior exams with calcifications related to cancer
Prior to screen-detected cancer	744	222	118
Prior to interval cancer	413	212	59
**Total number of prior exams**	**1157**	**434**	**177**

Table 2 summarizes the invasive status of the cancers. Of the 148 screen-detected cancers detected with calcifications, but without visible calcifications on the prior exam, 77 (52.4%) were invasive. Of the screen-detected cancers with calcifications visible in the prior exams, 71 (60.2%) were invasive when they were detected in the following screening round (i.e., two years later). Of the interval cancers with visible calcifications in the negative prior mammogram, 50 (84.7%) were invasive once they were detected. Overall, of all the 177 detectable calcifications associated with cancer from the prior exams, 121 (68.4%) developed into an invasive disease.

**Table 2:** Distribution of invasive and non-invasive cancers for all screen-detected calcifications and for all cancers with calcifications detectable in exams prior to screen-detected or interval cancer diagnosis

Distribution of invasive and non-invasive cancers	Total number of detectable cancers	Invasive	Non-invasive
Screen-detected calcifications	148	77 (52.4%)	71 (47.6%)

Earlier-detectable calcifications associated with cancer, prior to	Total number of detectable cancers	Invasive	Non-invasive
Screen-detected malignancies	118	71 (60.2%)	47 (39.8%)
Interval cancers	59	50 (84.7%)	9 (15.3%)
**Total earlier-detectable cancers**	**177**	**121 (68.4%)**	**58 (31.6%)**

The tumor stage for all invasive cancers with detectable calcifications is shown in Table 3. Of the screen-detected cancers detected by calcifications with negative prior exams, 22.1% were stage T2 or T3. For the invasive cancers detectable by calcifications on the exam prior to a recall or prior to an interval cancer, the percentage at tumor stage T2 and T3 were 25.3 and 40.0%, respectively. For all cancers that could have been detected earlier from their associated calcifications, the percentage of invasive cancers at tumor stage T2 or T3 was 31.4%. The tumor stage was not available for 11 invasive cancers.

**Table 3:** Distribution of the tumor stages for invasive cancers with calcifications

Distribution of tumor stages	Total number of invasive cancers	Stage T1 (< 2 cm)	Stage T2 (2–5 cm)	Stage T3 (> 5 cm)	Unknown
Screen-detected calcifications	77	59 (76.6%)	15 (19.5%)	2 (2.6%)	1 (1.3%)

Earlier-detectable calcifications associated with cancer, prior to:	Total number of invasive cancers	Stage T1 (< 2 cm)	Stage T2 (2–5 cm)	Stage T3 (> 5 cm)	Unknown
Screen-detected malignancies	71	51 (71.8%)	17 (23.9%)	1 (1.4%)	2 (2.8%)
Interval cancers	50	22 (44.0%)	18 (36.0%)	2 (4.0%)	8 (16.0%)
**Total earlier-detectable cancers**	**121**	**73 (60.3%)**	**35 (28.9%)**	**3 (2.5%)**	**10 (8.3%)**

## Discussion

In this study, we determined the sensitivity of a population-based screening program with double reading for calcifications associated with breast cancer using digital mammography. By considering all detectable malignant calcifications visible in exams prior to a screen-detected or an interval cancer diagnosis, we found that the screening sensitivity for malignant calcifications in the studied program was only 45.5%, while the specificity for calcifications was 99.5%. Because double reading is practiced in the screening program, we believe that it is unlikely that the generally low sensitivity is caused by the oversight of the radiologists; instead, it is more likely that these results reflect a high threshold in the judgment of the radiologists when characterizing calcifications as suspicious or unimportant.

This high threshold for recalling calcifications as a strategy to minimize overdiagnosis should perhaps be revised. We found that 68.4% of the women with cancer who had calcifications in a negative prior screening mammogram had developed an invasive cancer by the time it was detected. This could suggest that lowering of the threshold for recall in the national screening program is justifiable because more invasive cancers could be detected earlier. The frequency of invasive disease in women recalled with calcifications that were not detectable in prior images was 52.4%.

This finding indicates that in screening programs with a low recall rate, earlier detection of the calcifications visible in prior exams might prevent up to 16% of cancers from becoming invasive. Earlier detection would also reduce the occurrence of more advanced cancers; 31.4% of the invasive cancers with calcifications detectable on a prior exam presented as a stage T2 or T3 disease, i.e., the cancer was larger than 20 mm, at the time of diagnosis, compared to 22% when no calcifications were present in the prior exam.

In this study, we found that 54.5% of the screen-detected and interval cancers were detectable by calcifications in exams prior to diagnosis. In previous studies, it was found that 31–67% of screen-detected cancers could have been identified in earlier mammograms and 10–31% of the interval cancers were visible in prior screenings [17–28]. In our study, the percentage of cancer-related calcifications detectable on an exam prior to the later diagnosis of screen-detected cancers was 44, and 14% of the exams prior to interval cancer diagnoses contained detectable calcifications related to the cancer. These percentages are within the ranges reported in the literature; however, since previous studies did not make a distinction between calcifications and soft tissue lesions when calculating the number of cancers that could have been detected earlier, the results cannot be compared directly. Moreover, most previous studies were performed with screen-film mammography rather than digital mammography, which can have a different effect on the recall and cancer detection rates, especially for recalls based on calcifications [6, 30].

We constructed the ground truth by applying a CAD system for the detection of calcifications in the prior mammograms, followed by visual inspection by an experienced radiologist to determine the presence of calcifications related to the cancer detected later. The main purpose of using CAD was to reduce the workload of the radiologist and to make his judgment more objective. Because the CAD system was very sensitive, one could argue that use of a CAD system in screening could improve detection; however, current commercial CAD systems only provide mark regions for further attention to avoid calcifications being overlooked. For the detection of all calcifications in the ground truth, the specificity of the CAD system was only 51% when applied to the whole screening database and for the setting at which it was used. It should be noted that this specificity was achieved by considering all CAD marks irrespective of their scores; therefore, increasing the threshold on these scores could increase the specificity of the CAD system but reduce its maximum sensitivity. While this setting may be appropriate for use of CAD as a perception aid, it leaves the difficult problem of deciding which women with calcifications the radiologists should recall. To increase the role of CAD in calcification characterization algorithms, these systems should be developed to find an acceptable balance between sensitivity and specificity that would best help radiologists to stratify calcifications by risk. Previous studies have already demonstrated that CAD algorithms outperform radiologists in this task and there is potential to improve them considerably using new machine learning techniques [32, 33, 35–41].

A limitation of this study is that we do not know how many negative exams did contain calcifications. Negative exams will contain many benign calcifications and most likely also some malignant calcifications that did not yet result in a diagnosis of cancer within the two-year follow-up period we used for verification. Sometimes, benign calcifications are categorized as BI-RADS 2, but they are not always reported. It would be interesting to study how often benign calcifications occur that look suspicious but were not recalled, and to compare them to the malignant calcifications that were missed in screening. However, in this study, visual assessment of the large number of negative exams was not performed. Therefore, we cannot assess to what extent a higher recall of suspicious calcifications would lead to a strong increase of false positives.

Another limitation of our study is that we did not have access to information of all interval cancers in the period between 2013 and 2014. The absence of these cases and the exclusion of 60 cases with multiple recalls may have had a small effect on the results we present. The missing interval cancer information, as well as the absence of the radiology reports for interval cancers, can only lead to an underestimation of the number of detectable malignant calcifications and, due to this, the reported sensitivity may be slightly overestimated. Another limitation of our study is that it is based on data from one Dutch screening center, which may not be representative of other breast cancer screening programs. In particular, the radiologists in the center operated at a low recall rate, following the Dutch national breast cancer screening policy. Within Europe, the recall rate varies from 2 to 6% [16], with the screening program in the Netherlands operating at a recall rate of around 2.5%. In the United States, recall rates are substantially higher [42]. It is noted, however, that the interval cancer rate in the Dutch program and the percentage of cancers visible on prior mammograms are similar to those reported in the literature [17–28, 43]. This shows that our study data are representative of other screening practices.

To conclude, 54.5% of calcifications associated with cancer could potentially be detected earlier and this may substantially reduce the occurrence of invasive cancers in the screened population. It is therefore important to develop techniques that allow the earlier recall of patients with calcifications without increasing false positives and invasive diagnostic procedures to unacceptable levels.
